# AT‐RvD1 combined with DEX is highly effective in treating TNF‐*α*‐mediated disruption of the salivary gland epithelium

**DOI:** 10.14814/phy2.12990

**Published:** 2016-09-30

**Authors:** Justin T. Easley, Christina L. M. Maruyama, Ching‐Shuen Wang, Olga J. Baker

**Affiliations:** ^1^ School of DentistryUniversity of UtahSalt Lake CityUtah

**Keywords:** ALX/FPR2, AT‐RvD1, RvD1, salivary glands, Sjögren's syndrome

## Abstract

Sjögren's syndrome (SS) is an autoimmune disorder characterized by chronic inflammation and destruction of salivary and lacrimal glands leading to dry mouth and dry eyes, respectively. Currently, the etiology of SS is unknown and the current therapies have no permanent benefit; therefore, new approaches are necessary to effectively treat this condition. Resolvins are highly potent endogenous lipid mediators that are synthesized during the resolution of inflammation to restore tissue homeostasis. Previous studies indicate that the resolvin family member, RvD1, binds to the ALX/FPR2 receptor to block inflammatory signals caused by tumor necrosis factor‐alpha (TNF‐*α*) in the salivary epithelium. More recently, the corticosteroid, dexamethasone (DEX), was shown to be effective in reducing salivary gland inflammation. However, DEX, as with other corticosteroids, elicits adverse secondary effects that could be ameliorated when used in smaller doses. Therefore, we investigated whether the more stable aspirin‐triggered (AT) epimer, AT‐RvD1, combined with reduced doses of DEX is effective in treating TNF‐*α*‐mediated disruption of polarized rat parotid gland (Par‐C10) epithelial cell clusters. Our results indicate that AT‐RvD1 and DEX individually reduced TNF‐*α*‐mediated alteration in the salivary epithelium (*i.e*., maintained cell cluster formation, increased lumen size, reduced apoptosis, and preserved cell survival signaling responses) as compared to untreated cells. Furthermore, AT‐RvD1 combined with a reduced dose of DEX produced stronger responses (*i.e.,* robust salivary cell cluster formation, larger lumen sizes, further reduced apoptosis, and sustained survival signaling responses) as compared to those observed with individual treatments. These studies demonstrate that AT‐RvD1 combined with DEX is highly effective in treating TNF‐*α*‐mediated disruption of salivary gland epithelium.

## Introduction

Sjögren's syndrome (SS) is an autoimmune disease characterized by chronic inflammation and destruction of the salivary and lacrimal glands, leading to xerostomia and keratoconjunctivitis sicca (Miranda‐Rius et al. 2015). The pathophysiology of SS has been associated with elevated levels of proinflammatory cytokines, such as tumor necrosis factor‐*α* (TNF‐*α*), interferon‐*γ* (IFN‐*γ*), and interleukins (IL)‐1*β*, ‐6, ‐18, and ‐12 (Oxholm et al. 1992; Fox et al. 1994; Boumba et al. 1995; Ohyama et al. 1996; Halse et al. 1999; Eriksson et al. 2004; Manoussakis et al. 2007).

TNF‐*α* is mainly produced by macrophages and lymphocytes, but is also produced in salivary epithelial cells (Sisto et al. 2009). Several studies indicate that TNF‐*α* is likely to play a role in the pathogenesis of SS; for instance, TNF‐*α* is upregulated in both plasma and salivary glands of patients with SS and major salivary glands of animal models of SS (Humphreys‐Beher et al. 1994, 1998; Cha et al. 2002a,b; Nguyen et al. 2007; Sisto et al. 2009). Furthermore, neutralization of TNF‐*α* in non‐obese diabetic (NOD) mice by transgenic expression of soluble TNF‐receptor (p55) significantly reduced lymphocytic infiltration in submandibular and lacrimal glands associated with the decreased expression of the cell adhesion molecules, VCAM‐1 and ICAM‐1 (Hunger et al. 1996). Additionally, several in vitro studies indicate that TNF‐*α* causes apoptosis and disruption of tight junction integrity in the salivary epithelium which is necessary to drive saliva secretion (Baker et al. 2008; Nelson et al. 2014). However, clinical studies showed that neither etanercept nor infliximab (both therapies directed toward blocking TNF‐*α* signaling) improved SS in controlled clinical trials (Mariette et al. 2004; Sankar et al. 2004). A concrete rationalization of these observations is still lacking, however, a successive study suggested that unexpected biological effects of TNF‐*α* antagonists may have contributed to this effect due to an unanticipated elevation of TNF‐*α* observed following treatment with etanercept (Moutsopoulos et al. 2008). Currently, there are no treatments to decrease or eliminate lymphocytic infiltration observed in salivary glands with SS. We believe that understanding the mechanisms by which this cytokine works in vitro is a necessary step for future translational studies.

Our understanding of the endogenous mechanisms that govern natural resolution of inflammation is limited in salivary glands (where proresolution pathways that promote epithelial healing are under current investigation) (Odusanwo et al. 2012; Leigh et al. 2014; Nelson et al. 2014). Previous studies demonstrated that human and animal cells convert *ω*‐3 polyunsaturated fatty acids (PUFAs) into resolvins (Rv), which are highly potent, anti‐inflammatory agents controlling the duration and magnitude of inflammation in models of inflammation (Serhan et al. 2015). The synthetic pathways and molecular mechanisms of Rv are similar to lipoxins (LX), the well‐documented anti‐inflammatory lipid mediators. The Rv include (1) Rv of the E series (RvE1‐2), derived from eicosapentaenoic acid (EPA); and (2) Rv of the D series (RvD1, RvD2, RvD3, RvD4, and RvD5) and their aspirin‐triggered epimeric forms (AT‐RvD1‐6), derived from docosahexaenoic acid (DHA) (Serhan 2008; Serhan and Chiang 2008; Serhan et al. 2008). The LX include (1) LXA_4_; (2) LXB_4_; and (3) the LX epimer aspirin‐triggered‐15‐epi‐LXA_4_ (ATL), all derived enzymatically from arachidonic acid (AA) (Serhan et al. 1984, 1987; Serhan 1997).

Resolvin D1 (RvD1), specifically, was initially identified in inflammatory exudates from murine peritonitis (Serhan et al. 2000). RvD1 biosynthesis, structure, and stereochemistry have been elucidated and well established (Sun et al. 2007). Low‐dose aspirin treatment triggers the formation of aspirin‐triggered biosynthetic pathways (Sun et al. 2007). In the case of RvD1, the aspirin‐triggered routes lead to the biosynthesis of AT‐RvD1, which appears to be more resistant to rapid enzymatic degradation as compared to RvD1 (Sun et al. 2007). Our previous studies indicate that RvD1 and AT‐RvD1 reduce TNF‐*α*‐mediated disruption of epithelial integrity in the rat parotid Par‐C10 cell line as well as in mouse submandibular gland (SMG) cells (Odusanwo et al. 2012; Nelson et al. 2014). Additionally, both RvD1 and AT‐RvD1 bind to the seven transmembrane receptor, ALX/FPR2, to activate signaling pathways that enhance epithelial integrity and improve cell survival in the salivary epithelium (Odusanwo et al. 2012; Nelson et al. 2014). Moreover, RvD1 biosynthetic pathways are expressed and functional in the human and mouse salivary epithelium (Leigh et al. 2014).

Glucocorticoids, such as dexamethasone (DEX), bind to the intracellular glucocorticoid receptor *α*, thereby promoting dissociation from heat shock protein 90 and translocation to the nucleus (Patel et al. 2014). Consequently, they activate transcription factors that induce expression of anti‐inflammatory genes (Vandevyver et al. 2013). DEX has been used to treat a broad range of inflammatory diseases, including Crohn's disease (Cully 2015), rheumatoid arthritis (Fogel et al. 2015), noninfectious uveitis (Tomkins‐Netzer et al. 2014), and asthma (Gries et al. 2000), among other conditions. Alternatively, glucocorticoids cause repression of genes encoding proinflammatory mediators, including cytokines, chemokines, and inflammatory adhesion molecules (van der Velden 1998). This effect is mostly due to protein–protein interactions between the glucocorticoid–glucocorticoid receptor complex and the transcription factors, NF‐*κ*B and AP‐1. Glucocorticoids can inhibit upstream proinflammatory signaling through activation of both the NF‐*κ*B and MAPK signaling pathways (King et al. 2009). In salivary glands, corticosteroids have been used as irrigation agents in patients with SS, eliciting significant improvement in saliva flow rate (Izumi et al. 1998). However, the molecular mechanisms behind these effects are highly complex and not completely understood. In fact, corticosteroids commonly exhibit numerous systemic side effects when used in the current doses, such as hypertension (Lifton et al. 2001), weight gain (Van Dongen‐Melman et al. 1995), loss of sleep (Hinds et al. 2007), agitation (Vardy et al. 2006), swelling (Hempen et al. 2002), acne (Zouboulis and Piquero‐Martin 2003), and osteoporosis (Walsh et al. 2001). Furthermore, long‐term administration of glucocorticoids causes insulin resistance, which is associated with reduced parotid and SMG cell volume and diminished secretory function, indicating a deleterious effect on salivary gland homeostasis (Bighetti et al. 2014).

A recent pilot study treated NOD/ShiLtJ SS‐like mice intravenously with AT‐RvD1 (0.01–0.1 mg/kg) or DEX (4.125–8.25 mg/kg) twice a week for 14 weeks beginning at 4 weeks of age before disease onset (Easley et al. 2015). Results demonstrated that DEX treatment prevents lymphocytic infiltration into the SMG while AT‐RvD1 treatment alone did not significantly affect lymphocytic infiltration into the SMG. Interestingly, both AT‐RvD1 and DEX caused downregulation of SS‐associated inflammatory genes and reduction of apoptosis (Easley et al. 2015).

The goal of this study was to determine whether a combination of DEX and AT‐RvD1 is effective in treating the damaging effects of proinflammatory cytokine signaling in vitro for subsequent in vivo applications. Our findings indicate that AT‐RvD1 and DEX individually reduced TNF‐*α*‐mediated alteration of the salivary epithelium (*i.e*., maintained cell cluster formation, lumen size, decreased apoptosis, and maintained survival signaling responses) as compared to untreated cells. Furthermore, AT‐RvD1 combined with a reduced dose of DEX produced even stronger responses (*i.e*., more robust cell cluster formation, larger lumen size, further reduced apoptosis, and sustained signaling responses) as compared to those observed with individual treatments. These results indicate that AT‐RvD1 combined with reduced doses of DEX is highly effective in treating TNF‐*α*‐mediated disruption of the salivary gland epithelium.

## Materials and Methods

### Par‐C10 culture and treatment

A quantity of 100 *μ*l of growth factor‐reduced Matrigel (GFR‐MG) (8 mg/mL; 2:1 GFR‐MG: DMEM‐Ham's F12 [1:1] medium; Becton Dickinson, Franklin Lakes, NJ) was allowed to solidify in a 37°C incubator for 1 h in eight‐well chambers mounted on No. 1.5 German borosilicate coverglass (Nalge Nunc International, Naperville, IL). Then, Par‐C10 cells (15,000 cells/well; passages 30–60) were plated on the GFR‐MG in DMEM‐Ham's F12 (1:1) medium with supplements and treated with TNF‐*α* (100 ng/mL; Becton Dickinson), then incubated for 1 h or 6 h at 37°C. Cells were then treated with AT‐RvD1 (100 ng/mL; Cayman Chemical, Ann‐Arbor, MI), and varying doses of DEX (25–100 ng/mL). Three‐dimensional Par‐C10 cell clusters were used for assays after incubation at 37°C with 95% air and 5% CO_2_ for 72 h.

### Cell cluster quantification

Par‐C10 salivary cell clusters were counted using a Leica DMI6000B microscope (Leica Microsystems, Mannheim, Germany) with the 10× objective to render a field of view of 1000 × 700 *μ*m. We counted cell clusters from three different positions within each well (top‐left, middle, and bottom‐right). In order to calculate the number of cell clusters/mm^2^, we averaged the number of cell clusters from each of the three positions and divided it by the total field area (0.85 *μ*m^2^).

### Fluorescence microscopy analysis

Par‐C10 cell clusters grown on GFR‐MG were fixed in 4% paraformaldehyde for 10 min at room temperature, incubated with 0.1% Triton X‐100 in PBS for 5 min, and washed three times with PBS. The cells were then incubated with 5% goat serum for 1 h at room temperature and washed three times with PBS. This was followed by incubation overnight at 4°C with rabbit anti‐ZO‐1 (Invitrogen, Carlsbad, CA) at 1:200 dilution in 5% goat serum. The next day, cells were allowed to warm up to room temperature for 20 min and washed three times for 5 min with PBS. Next, cells were incubated for 1 h with Alexa Fluor 488‐conjugated goat anti‐rabbit (1:500 dilution in 5% goat serum) and washed three times with 1 PBS. Finally, cells were stained for 1 h with phalloidin to localize filamentous actin (1:400 dilution in PBS; Sigma Aldrich, St. Louis, MO), and 5 min with TO‐PRO‐3 iodide (1:10,000 dilution in PBS; Thermo‐Scientific, Waltham, MA) to visualize nuclei. Images were obtained using a Carl Zeiss 710 confocal microscope (Carl Zeiss, Oberkochen, Germany). All images were analyzed using ZEN 2012 (Blue Edition; Carl Zeiss) and Graphpad Prism 6 (GraphPad Software, Inc. La Jolla, CA) software. Lumen sizes (diameter of the lumen) were measured at the maximum diameter of 10–15 cell clusters per experimental group.

### TUNEL analysis

Par‐C10 cells (passages 52–57) were plated on GFR‐MG as described above and preincubated with TNF‐*α* for 1 h. Cells were then treated with AT‐RvD1 (100 ng/mL) and varying doses of DEX (25–100 ng/mL) both alone and in combination for 72 h. A terminal deoxynucelotidyl transferase‐mediated dUTP nick end‐labeling (TUNEL) assay was performed with a Click‐It^®^ TUNEL Alexa Fluor^®^ 488 imaging assay (Life Technologies, Grand Island, NY) as described previously (Easley et al. 2015). Following fixation and permeabilization steps, some wells were incubated with DNase for positive control data. Maximum intensity projections of each well were produced by creating 4 × 4 tile images (20× objective; 1280 × 1280 *μ*m) of four‐section *z*‐stacks illustrating colocalization of the TOPRO‐3 Iodide^®^ nuclear stain and TUNEL AlexaFluor^®^ 488 expression (showing apoptotic cells). Laser intensity and gain settings for TUNEL AlexaFluor^®^ 488 were kept consistent across all samples imaged. ZEN (Carl Zeiss), Prism (Graphpad), and Photoshop (Adobe Systems, San Jose, CA) software were utilized to process and analyze the percentage of TUNEL‐positive cells among cell clusters.

### Western Blot analysis

Cells were lysed in 100 *μ*L of 5× Laemmli buffer (120 mmol/L Tris‐HCl, pH 6.8, 10% (v/v) glycerol, 2% (w/v) SDS, 1 mmol/L dithiothreitol, and 0.002% (w/v) bromophenol blue). Lysates were sonicated for 5 sec with a sonic sismembrator (model 120; microtip; amplification 45%; Fisher Scientific, Pittsburgh, PA) and boiled for 5 min at 95°C. Cell lysates were subjected to electrophoresis in 4–15% (w/v) Mini‐Protean TGX gels (Bio‐Rad, Hercules, CA) and transferred to nitrocellulose membranes. Membranes were blocked for 1 h with 3% (w/v) bovine serum albumin (BSA) in Tris‐buffered saline (0.137 mol/L NaCl, 0.025 mol/L Tris (hydroxymethyl)‐aminomethane, pH 7.4) containing 0.1% (v/v) Tween‐20 (TBST) and immunoblotted overnight with primary antibody at 4°C in TBST containing 3% (w/v) BSA. The following antibody was utilized: rabbit anti phospho‐Akt that recognizes endogenous phospho‐Akt when phosphorylated at Ser473 (1:1000 dilution; Cell Signaling Technology, Danvers, MA). After incubation with the primary antibody, membranes were washed three times for 15 min each with TBST and incubated with peroxidase‐linked goat anti‐rabbit IgG antibody (1:2000 dilution; Cell Signaling Technology) at room temperature for 1 h. The membranes were washed three times for 15 min each with TBST, treated with Clarity chemiluminescence detection reagent (Bio‐Rad), and protein bands were visualized using a Chemi‐Doc MP Imager (Bio‐Rad). Quantification of the bands was performed using Image Lab (Bio‐Rad; release 4.1). For signal normalization, membranes were treated with Restore stripping buffer (Thermo‐Scientific) and reprobed with rabbit anti‐Pan‐Akt (1:1000 dilution; Cell Signaling Technology) which detects endogenous levels of total analogous protein regardless of post‐translational modifications. Samples for phosphorylation studies were collected at 0 min after treatment, as well as 5, 15, 30, and 60 min after treatment in order to visualize changes in phosphorylation (that occur on a markedly shorter time scale than that used for other experiments; see previous study (Odusanwo et al. 2012)).

### Statistics

Both one‐way ANOVAs (Dunnett's test) and *t‐*tests were run on data sets to determine statistical significance.

## Results

### Treatment with AT‐RvD1 and DEX alone and in combination enhances Par‐C10 cell cluster growth

**Figure 1:**
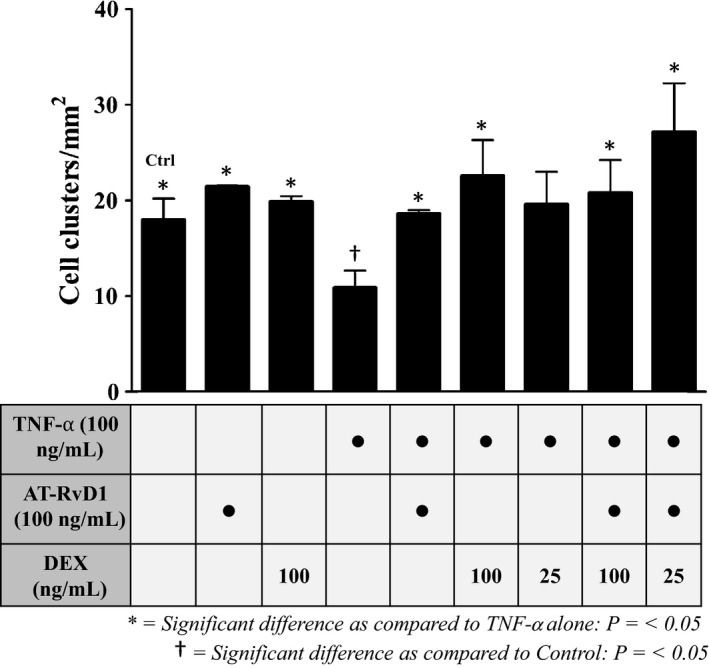
Treatment with aspirin‐triggered resolvin D1 (AT‐RvD1) and dexamethasone (DEX) alone and in combination enhances Par‐C10 cell cluster growth on growth factor‐reduced Matrigel (GFR‐MG). Par‐C10 cells were grown on GFR‐MG in eight‐well chambers as described in Materials and Methods and preincubated in the absence or presence of TNF‐*α* (100 ng/mL for 1 h), then treated with AT‐RvD1 (100 ng/mL) and DEX (25–100 ng/mL) alone and in combination. Cell clusters were then quantified using light microscopy. Cell cluster growth was quantified and expressed as means ± SE of results from 3 or more experiments, where *P < 0.05 indicate significant differences from cells incubated with TNF‐*α* alone, and ^†^ *P < *0.05 indicate significant differences from control (untreated) cells.

Par‐C10 cells were plated on GFR‐MG and preincubated with TNF‐*α* followed by treatment with AT‐RvD1 and DEX alone and in combination as described in Materials and Methods. Then, salivary cell clusters were quantified using light microscopy as described in Materials and Methods. As shown in Figure 1, untreated control cells as well as cells subjected to single treatments with AT‐RvD1 (100 ng/mL) or DEX (100 ng/mL) for 72 h showed increased salivary cell cluster growth as compared to cells treated with TNF‐*α* alone (100 ng/mL). Additionally, cells that were preincubated with TNF‐*α* (100 ng/mL) for 1 h followed by treatment with AT‐RvD1 (100 ng/mL) and DEX (25–100 ng/mL) alone and in combination recovered the number of salivary cell clusters after 72 h. Surprisingly, cells preincubated with TNF‐*α* for 1 h and then treated with AT‐RvD1 (100 ng/mL) and ¼ dose of DEX (25 ng/mL) for 72 h displayed a significant increase in the number of cell clusters grown as compared to cells treated with TNF‐*α* alone. Note that only cells preincubated with TNF‐*α* without any subsequent treatment showed a statistically significant cell cluster reduction from the control group.

### Treatment with AT‐RvD1 and DEX alone and in combination enhances Par‐C10 cell cluster formation

**Figure 2:**
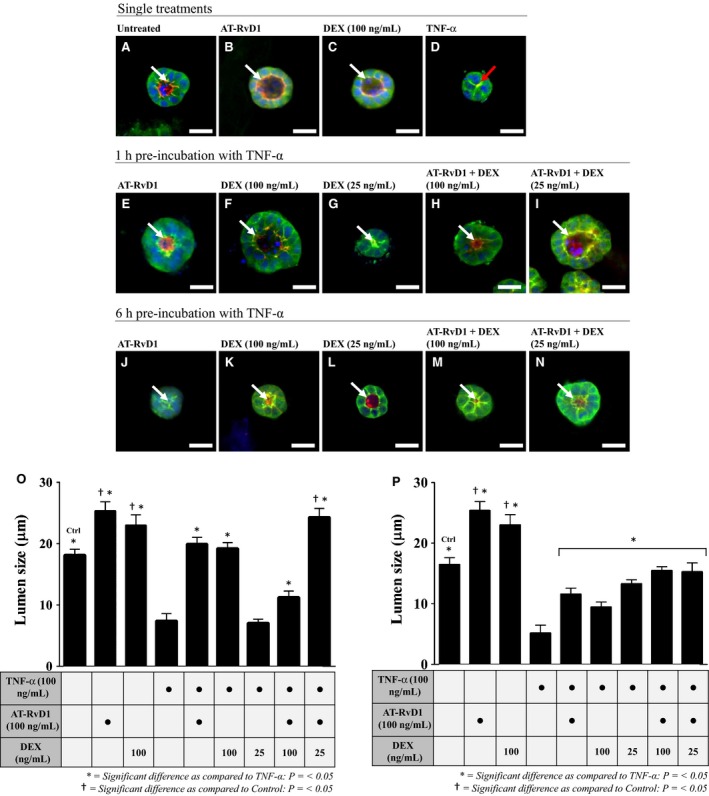
Treatment with aspirin‐triggered resolvin D1 (AT‐RvD1) and dexamethasone (DEX) alone and in combination enhances cell cluster and lumen formation in Par‐C10 cells grown on growth factor‐reduced Matrigel (GFR‐MG). To establish baseline data, Par‐C10 cells were subjected to single treatments (A–D). Next, cells were preincubated with TNF‐*α* (100 ng/mL) for 1 h (E–I) or 6 h (J–N) and treated for 72 h with AT‐RvD1(100 ng/mL) and DEX (25–100 ng/mL) alone and in combination. Then, cell clusters were fixed with 4% of paraformaldehyde and stained with rabbit anti‐ZO‐1 and Alexa Fluor^®^ 488 (green), followed by the F‐actin stain Alexa Fluor^®^ phalloidin 568 (red) and the nuclear stain TO‐PRO ^®^‐3 iodide (blue). White arrows point to apical localities of ZO‐1 (2A‐C, E‐N), while the red arrow shows an improper collapsed lumen in cells treated with TNF‐*α* (2D). Images were obtained and analyzed using a Carl Zeiss 710 confocal microscope and ZEN software. All scale bars represent 20 *μ*m. Lumen sizes were measured following either a 1 h (O) or 6 h (P) preincubation with TNF‐*α*, followed by 72 h treatments with AT‐RvD1 (100 ng/mL) and DEX (25–100 ng/mL) alone and in combination. Data are expressed as means ± SE of results from 3 or more experiments, where *P < 0.05 indicate significant differences from cells incubated with TNF‐*α* alone and ^†^ *P < *0.05 indicate significant differences from control (untreated) cells.

Par‐C10 cells were plated on GFR‐MG and preincubated with TNF‐*α* followed by treatment with AT‐RvD1 and DEX alone and in combination as described in Materials and Methods. Then, cells were then stained and visualized under a fluorescence microscope as described in Materials and Methods. As shown in Figure 2A–C, the control and single treatment cells (*i.e.,* untreated, AT‐RvD1 (100 ng/mL) alone, and DEX (100 ng/mL) alone) grown for 72 h displayed apically localized ZO‐1 and F‐actin ring (white arrows), indicating polarization and formation of hollow lumens. In contrast, cells incubated with TNF‐*α* (100 ng/mL) alone for 72 h lacked both apically localized ZO‐1 and an F‐actin ring (Fig. 2D; red arrow), indicating loss of cell polarity and lumen formation, typical of cells grown under this condition (Odusanwo et al. 2012). After preincubation with TNF‐*α* (100 ng/mL) for 1 h, treatments with AT‐RvD1 (100 ng/mL) or DEX (25–100 ng/mL) alone and in combination for 72 h recovered lumen formation (Fig. 2E–I) as compared with cells treated with TNF‐*α* alone. Strikingly, cells preincubated with TNF‐*α* for 1 h, then treated with AT‐RvD1 (100 ng/mL) and ¼ dose of DEX (25 ng/mL) (Fig. 2I) displayed highly organized apical ZO‐1 and F‐actin rings, indicating well‐developed cell polarity and formation of defined lumens as compared to cells grown under all other conditions. Similar results were observed after Par‐C10 cells were preincubated with TNF‐*α* (100 ng/mL) for 6 h and treated with AT‐RvD1 and DEX both alone and in combination (Fig. 2J–N).

Given the results observed using fluorescence microscopy, we decided to quantify the effects of treatment with AT‐RvD1 and DEX alone and in combination on the formation of apical lumen structures after preincubation with TNF‐*α* (100 ng/mL) for 1 h (Fig. 2O) and 6 h (Fig. 2P). Lumen sizes were measured as described in Materials and Methods. As shown in Figure 2O, control and single treatment cells (*i.e*. untreated, AT‐RvD1 (100 ng/mL) or DEX (100 ng/mL) alone) grown for 72 h exhibited significantly larger lumens than cells preincubated with TNF‐*α* for 1 h. Furthermore, cells preincubated with TNF‐*α* for 1 h and treated with AT‐RvD1 (100 ng/mL) and DEX (25–100 ng/mL) alone and in combination for 72 h displayed significantly larger lumens than cells incubated with TNF‐*α* alone in all conditions except when treated with DEX (25 ng/mL) alone. Surprisingly, cells preincubated with TNF‐*α* for 1 h and treated with AT‐RvD1 (100 ng/mL) and ¼ dose of DEX (25 ng/mL) for 72 h exhibited significantly larger lumens as compared with both untreated control cells as well as cells preincubated with TNF‐*α* for 1 h and treated with AT‐RvD1 (100 ng/mL) or full doses of DEX (100 ng/mL) alone for 72 h. As shown in Figure 2P, cells preincubated with TNF‐*α* for 6 h and treated with AT‐RvD1 (100 ng/mL) and DEX (25–100 ng/mL) alone and in combination for 72 h displayed significantly larger lumens in all treatment groups compared with cells incubated with TNF‐*α* alone, although lumen size was not as large compared with treated cells preincubated with TNF‐*α* for 1 h (Fig. 2O).

### Treatment with AT‐RvD1 and DEX alone and in combination mitigates TNF‐α mediated cellular apoptosis

**Figure 3:**
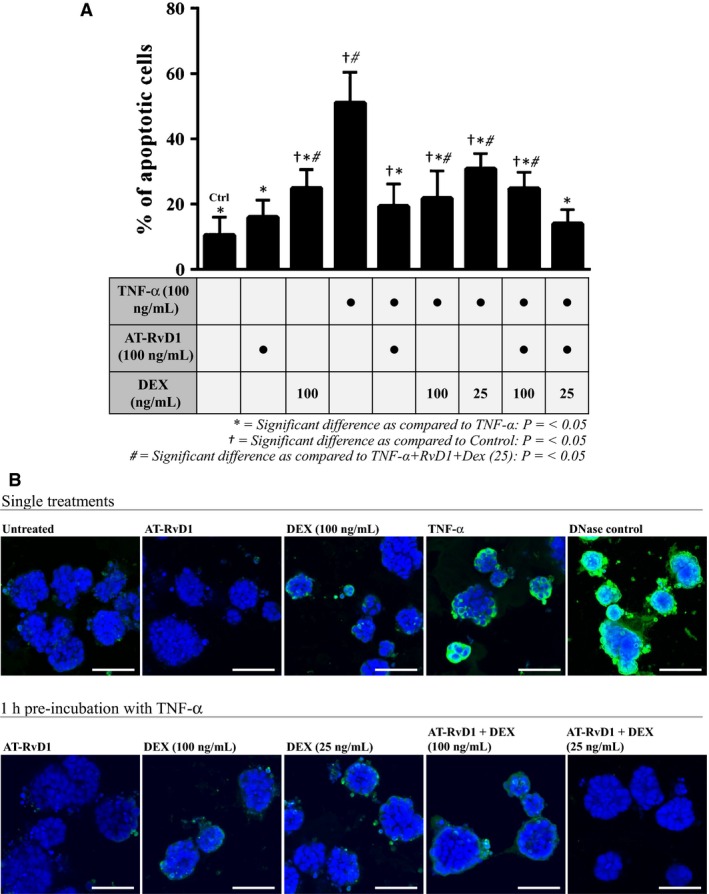
Treatment with aspirin‐triggered RvD1 (AT‐RvD1) and a reduced dose of dexamethasone (DEX) mitigates TNF‐*α* mediated apoptosis. Par‐C10 cells were grown on GFR‐MG in eight‐well chambered coverglass slides as described in Materials and Methods. Then, cells were preincubated in the absence or presence of TNF‐*α* (100 ng/mL for 1 h) and treated with AT‐RvD1 (100 ng/mL) and DEX (25–100 ng/mL) alone and in combination for 72 h. (A) Percentages of apoptotic cells in Par‐C10 cell clusters were determined by a terminal deoxynucleotidyl transferase‐mediated dUTP nick end‐labeling (TUNEL) assay. Data are expressed as means ± SE of results from 3 or more experiments, where *P < 0.05 indicate significant differences from cells incubated with TNF‐*α* alone, ^†^ *P < *0.05 indicate significant differences from control (untreated) cells, and ^#^ *P < *0.05 indicate significant differences from the combination treatment group consisting of AT‐RvD1 (100 ng/mL) and DEX (25 ng/mL) after 1 h preincubation with TNF‐*α*. (B) Representative images depicting varying degrees of apoptosis per treatment group. TOPRO‐3^®^ Iodide was used to visualize all nuclei (blue) and TUNEL Alexa Fluor^®^ 488 was used to visualize apoptosing cells (green). Scale bars represent 50 *μ*mol/L.

To assess the effects of AT‐RvD1 and DEX on cell death following preincubation with TNF‐*α*, we performed a TUNEL assay on the Par‐C10 cell clusters grown on GFR‐MG. As shown in Figure 3A and B, a basal level of TUNEL‐positive cells (indicating percentage of apoptotic cells) was determined using untreated (control) cells. Both single treatments with DEX (100 ng/mL) and TNF‐*α* (100 ng/mL) resulted in a significant increase in the degree of apoptosis from the untreated cells, although AT‐RvD1 alone (100 ng/mL) did not increase the percentage of apoptotic cells. After preincubation with TNF‐*α* for 1 h, AT‐RvD1 alone and in combination with DEX caused a significant reduction in apoptotic cells as compared to cells treated with TNF‐*α* alone (see asterisks). Interestingly, the treatment consisting of AT‐RvD1 (100 ng/mL) and a reduced dose of DEX (25 ng/mL) was the most effective in reducing apoptosis, with values similar to untreated cells. Figure 3B shows representative images of percentages of TUNEL‐positive cells in each treatment group.

### Treatment with AT‐RvD1 and DEX alone and in combination enhances Akt phosphorylation in Par‐C10 cells

**Figure 4:**
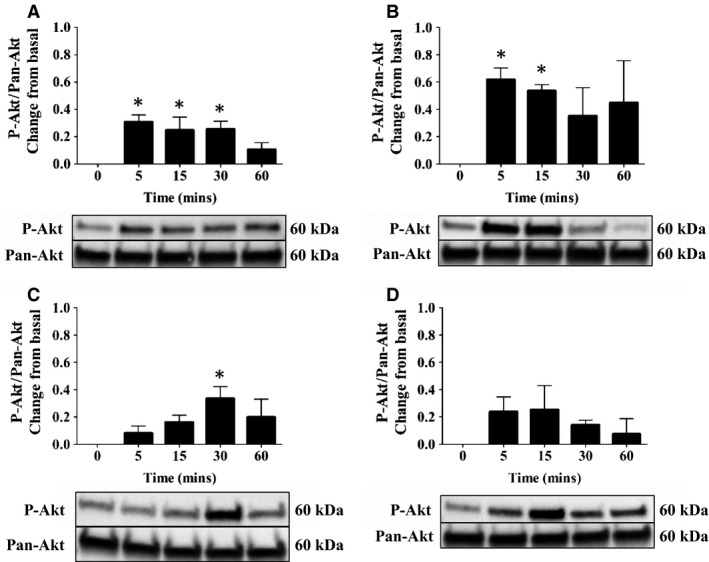
Aspirin‐triggered RvD1 (AT‐RvD1) and dexamethasone (DEX) alone and in combination cause transient phosphorylation of Akt in the salivary epithelium. Par‐C10 cells were incubated with (A) AT‐RvD1 (100 ng/mL), (B) DEX (100 ng/mL), (C) AT‐RvD1 (100 ng/mL) + DEX (25 ng/mL), and (D) TNF‐*α* (100 ng/mL) for 0–60 min. Cells were then lysed with Laemmli buffer, and the expression of phosphorylated Akt (p‐Akt) was detected by western blot analysis. Phosphorylated Akt values were normalized to total protein using a Pan Akt antibody. Data are expressed as means ± SE of results from 3 or more experiments. Significant differences in protein expression as compared to the basal value (0 min) are indicated by asterisks. Results from a representative experiment are shown below graphs.

To determine whether AT‐RvD1 or DEX promotes activation of cell survival signaling pathways, we measured Akt phosphorylation. For these studies, Akt phosphorylation was quantified by western blot analysis as described in Materials and Methods. As shown in Figure 4A, treatment with AT‐RvD1 (100 ng/mL) induced a slight yet sustained level of Akt phosphorylation from 5 to 60 min. Furthermore, treatment with DEX (100 ng/mL) induced a higher and sustained level of Akt phosphorylation starting at 5 min and continuing through 60 min as compared with AT‐RvD1 treatment (Fig. 4B). Moreover, treatment with a combination of AT‐RvD1 (100 ng/mL) and ¼ dose of DEX (25 ng/mL) caused sustained phosphorylation of Akt beginning at 5 min and continuing through 60 min with peak phosphorylation occurring at 30 min (Fig. 4C). In contrast, treatment with TNF‐*α* caused an initial phosphorylation of Akt at 5 min and transiently decreased phosphorylation levels to those near basal values after 60 min (Fig. 4D). Significant differences in protein expression as compared to the basal values (0 min) are indicated by asterisks.

## Discussion

This study demonstrates that single and combination treatments with AT‐RvD1 and DEX can help to mitigate the damaging effects of TNF‐*α* (*i.e*., decreased cell cluster number, distorted lumen formation and size, and increased cell death).

We observed that treatment with AT‐RvD1, both alone and in combination with DEX significantly improved Par‐C10 cell cluster growth on GFR‐MG after 1 h preincubation with TNF‐*α* (Fig. 1). These results are consistent with our previous findings showing that AT‐RvD1 enhances epithelial cell growth in Par‐C10 cell monolayers after incubation with this cytokine (Odusanwo et al. 2012). Additionally, we found that treatment with full doses of DEX significantly improved Par‐C10 cell cluster growth on GFR‐MG after a 1 h preincubation with TNF‐*α* (Fig. 1), which has not been studied previously. Furthermore, all drug treatments significantly decreased cell apoptosis (as determined by TUNEL‐positive cells; Fig. 3A and B) as compared with cells preincubated with TNF‐*α* without any subsequent treatment. The combination of AT‐RvD1 and a reduced dose of DEX produced the most striking decrease in apoptosis of all treatment groups in the presence of TNF‐*α* (*i.e*., the only treatment group to have statistically equivalent levels of apoptosis as the untreated cells), substantiating the premise that a combination treatment might produce the most robust results. In a previous study, we demonstrated that AT‐RvD1 causes Erk1/2 phosphorylation to block caspase‐3 activity in salivary epithelial cells (Nelson et al. 2014), further suggesting a proproliferation and antiapoptotic effect of AT‐RvD1 in the presence of TNF‐*α*.

Lumenization is important for salivary cells to differentiate into a secretory phenotype (Tucker 2007). We observed that cells preincubated with TNF‐*α* for 1 h then treated with a combination of AT‐RvD1 and ¼ dose of DEX exhibited a dramatic recovery of lumen formation (Fig. 2I and O) as compared to cells treated with TNF‐*α* alone (Fig. 2D and O). The recovery observed with all treatments in this study (Fig. 2O and P) is consistent with previous studies showing that cells treated with AT‐RvD1 recover lumen formation when grown on GFR‐MG in the presence of TNF‐*α* (Odusanwo et al. 2012). Together, these results indicate that treatment with AT‐RvD1 and DEX alone and in combination attenuate the disruption of salivary epithelium caused by TNF‐*α* while promoting lumen formation.

Activation of the phosphatidylinositol PI3k/Akt pathway has been shown to inhibit proapoptotic signals to promote survival in epithelial tissues (Kane et al. 1999; Yilmaz et al. 2004). Previous studies showed that Rv elicit phosphorylation of the Akt survival pathway in a time‐ and dose‐dependent manner (Ohira et al. 2010). Furthermore, diabetic mice treated with RvD1 showed enhanced activation of Akt in adipose tissue and vasculature, thereby reducing adipose tissue accumulation and improving insulin sensitivity (Hellmann et al. 2011). Additionally, treatment with AT‐RvD1 was shown to phosphorylate Akt in Par‐C10 cells, and the subsequent inhibition with Akt‐targeted siRNA resulted in a significant reduction in cell migration (Odusanwo et al. 2012). In the current study, Par‐C10 cells treated with AT‐RvD1 exhibited phosphorylation of Akt at serine residue 473 (Fig. 4A), suggesting the functionality of AT‐RvD1 signaling through the ALX/FPR2. All treatments with the exception of TNF‐*α* caused Akt phosphorylation, demonstrating that treatment with AT‐RvD1 and DEX alone and in combination is able to activate cell survival and proliferation pathways in salivary epithelium.

We cannot conclusively demonstrate why a combination of ¼ dose of DEX with AT‐RvD1 was more effective than a full dose of DEX with AT‐RvD1. However, we could speculate that DEX might elicit effects on the ALX/FPR2. ALX/FPR2 has various ligands including Annexin A1 (ANXA1; shown to be upregulated by DEX) (Bena et al. 2012; Yang et al. 2013), lipoxin A_4_ (LXA_4_), and RvD1. The binding sites for ANXA1 and LXA4 are well known. Specifically, ANXA1 binds to the N‐terminus (aa 1–39) and extracellular loop II (aa 146–240) of the ALX/FPR2 and LXA_4_ binds to extracellular loop III (aa 241–351) (Bena et al. 2012). However, the RvD1 binding site is unknown, although it has been suggested to share a common binding site with LXA_4_ through ligand competition assays (Chiang et al. 2006; Bozinovski et al. 2012; Cooray et al. 2013). As such, we speculate that RvD1 and ANXA1 could bind simultaneously to the ALX/FPR2, leading to receptor desensitization (Tilley and Rockman 2006). Therefore, it is possible that ¼ dose of DEX (resulting in a lower production of ANXA1) would allow for optimal activation of the ALX/FPR2, thus preventing the deleterious effects caused by TNF‐*α*.

In summary, our results indicate that AT‐RvD1 combined with a reduced dose of DEX is highly effective (*i.e.,* more robust cell cluster formation, larger lumen size, decreased cell death, and sustained signaling responses) in treating TNF‐*α*‐mediated disruption of the salivary gland epithelium. These findings offer promise for ultimately eradicating the detrimental side effects associated with DEX by using a safe and complementary drug that allows for a reduced dose of the corticosteroid, without compromising patient outcomes. Future studies will be directed toward testing this treatment in SS animal models for prospective use in clinical studies.

## Conflict of Interest

None declared.
